# Glaucoma

**Published:** 1885-08

**Authors:** A. W. Calhoun

**Affiliations:** Professor of Diseases of the Eye, Ear and Throat, in the Atlanta Medical College, Atlanta, Ga.


### Clinic Reports.

### GLAUCOMA.

REMARKS BY A. W. CALHOUN, M. D., PROFESSOR OF DISEASES OF THE EYE, EAR AND THROAT, IN THE ATLANTA MEDICAL COLLEGE, ATLANTA, GA.

Reported for THE ATLANTA MEDICAL AND SURGICAL JOURNAL.

A very common affection falling into the hands of general practitioners, and which too often results fatally to the vision, is glaucoma.

Pathologically speaking, the disease is an excavation of the optic nerve, produced by too much intraocular pressure, which, if it continues without relief, will result sooner or later in total blindness.

One of the first symptoms the patient experiences is the more or less rapid increased loss of sight. Most usually accompanying this diminution of sight is a severe neuralgic pain in and around the eye and down the side of the nose of the affected side. The pain is not unfrequently so great that the patient and the attending physician do not recognize the disease, supposing they have a severe type of neuralgia to deal with, but if the physician will examine the ball closely during the existence of the neuralgia, he will notice several points that will aid him in making his diagnosis. No matter how recent the attack, or how severe or slight the neuralgic pains, the vision immediately begins to fail, rapidly diminishing until blindness follows, unless the disease is checked.

The ball most usually becomes very hard, the patient often complaining of this sensation, but the hardness is not invariably an accompanying symptom of the disease. You will also notice that the superficial blood vessels are very much engorged, and that the anterior chamber with the pupil presents a more or less muddy appearance, due to lymph deposited in the aqueous humor. The books mention dilatation of the pupil as a prominent symptom of glaucoma, but this is so frequently not the case that it might be misleading to expect to find it. I constantly see cases of well marked glaucoma with the pupil normal in appearance and no undue tension of the ball. To decide upon these latter cases necessarily requires the use of the ophthalmoscope, but the ordinary cases are so pronounced that almost any observant physician upon close examination would be able to recognize it. The severe pain, the sudden blindness, the congested condition of the ball, the muddy appearance of the aqueous humor, and, if it exist, the enlargement of the pupil and the hardness of the ball would almost of necessity lead to the conclusion that your patient had an attack of glaucoma. Sometimes, notwithstanding the severity of such an attack, with all these symptoms, the patient will recover more or less vision, only in time to undergo another recurrent attack.

Just at this point I wish to warn you not to use atropia in suspected cases of glaucoma, for it will quickly develop any lurking attack of the disease. If there is no predisposition to the disease, the free and even long-continued use of it will have no influence whatever in bringing about the disease. After the disease is once set up it is only a question of time for entire loss of sight to occur. The disease never arrests itself. What we have said refers more especially to acute attacks. Other so-called chronic cases progress very slowly without pain, without inflammation, but with a steady diminution of sight with the other marked symptoms to total blindness.

Glaucoma usually occurs in but one eye, but it is only a question of time, as a rule, when it will develop in the other eye.

I have seen it in quite young persons, but you will most frequently find it in middle life, and, according to my observation, it is more frequent in females, occurring about the change of life.

TREATMENT.

I know of but one remedy for the disease. You cannot cure it, but you can arrest it just where you find it, and in some cases the vision will be almost normally restored. Should the patient become blind from the disease, and remain so even for a short time, there is no hope for restoration of sight. In such a case, if the severe pain should continue after total loss of sight, an operation will relieve it at once. The only remedy is a large iridectomy, cutting out a fourth or fifth of the iris, making the excision entire up to the periphery.

Formerly chloroform was used on account of the severity of the operation, but the discovery of cocaine does away with the trouble and danger of chloroform. A few drops of a sixth per cent. solution of the muriate of cocaine give almost complete immunity from pain.

After making the iridectomy, atropia can be used with great advantage, but let me urge you never to use it previous to the operation. In making the operation on the case before you, the incision is made at the upper corneo-sclerotic junction with a Graefe's cataract knife, the iris grasped at the pupillary margin and drawn out through the wound and cut off with iris scissors, cutting from one side of the wound to the other. The hemorrhage that takes place in this case in the anterior chamber amounts to but little, as it will be entirely absorbed in two or three days.

The cnly after-treatment we will give this patient is to dress the eye twice daily, using a two-grain solution of atropia as an eye wash and bandaging the eye. The patient will be kept in bed for four or five days.

In conclusion, gentlemen, allow me to advise you, if you are in doubt about your case being one of glaucoma, call in consultation your professional neighbor, for if you do not act promptly your patient will irretrievably lose the eye.

